# Molecular Insights into Carbapenem Resistance in *Klebsiella pneumoniae*: From Mobile Genetic Elements to Precision Diagnostics and Infection Control

**DOI:** 10.3390/ijms27031229

**Published:** 2026-01-26

**Authors:** Ayman Elbehiry, Eman Marzouk, Adil Abalkhail

**Affiliations:** Department of Public Health, College of Applied Medical Sciences, Qassim University, P.O. Box 6666, Buraydah 51452, Saudi Arabia; ar.elbehiry@qu.edu.sa (A.E.); e.marzouk@qu.edu.sa (E.M.)

**Keywords:** *Klebsiella pneumoniae*, carbapenem resistance, carbapenemases, antibiotic resistance, plasmids, mobile genetic elements, molecular diagnostics, gut microbiota

## Abstract

Carbapenem-resistant *Klebsiella pneumoniae* (CRKP) has become one of the most serious problems confronting modern healthcare, particularly in intensive care units where patients are highly susceptible, procedures are frequent, and antibiotic exposure is often prolonged. In this review, carbapenem resistance in *K. pneumoniae* is presented not as a fixed feature of individual bacteria, but as a process that is constantly changing and closely interconnected. We bring together evidence showing how the spread of successful bacterial lineages, the exchange of resistance genes, and gradual genetic adjustment combine to drive both the rapid spread and the long-lasting presence of resistance. A major focus is placed on mobile genetic elements, including commonly encountered plasmid backbones, transposons, and insertion sequences that carry carbapenemase genes such as *blaKPC*, *blaNDM*, and *blaOXA-48-like*. These elements allow resistance genes to move easily between bacteria and across different biological environments. The human gut plays a particularly important role in this process. Its microbial community serves as a largely unseen reservoir where resistance genes can circulate and accumulate well before infection becomes clinically apparent, making prevention and control more difficult. This review also discusses the key biological factors that shape resistance levels, including carbapenemase production, changes in the bacterial cell membrane, and systems that expel antibiotics from the cell, and explains how these features work together. Advances in molecular testing have made it possible to identify resistance more quickly, supporting earlier clinical decisions and infection control measures. Even so, current tests remain limited by narrow targets and may miss low-level carriage, hidden genetic reservoirs, or newly emerging resistance patterns. Finally, we look ahead to approaches that move beyond detection alone, emphasizing the need for integrated surveillance, thoughtful antibiotic use, and coordinated system-wide strategies to lessen the impact of CRKP.

## 1. Introduction

Intensive care units (ICUs) care for patients with severe illness who often require central lines, ventilators, and other invasive devices. These patients also receive broad-spectrum antibiotics for extended periods. This combination creates conditions that strongly favor the selection and spread of carbapenem-resistant *Klebsiella pneumoniae* (CRKP) [1,2]. Prolonged ICU stays, repeated courses of carbapenems and other antibiotics, and multiple invasive procedures are well-established risk factors for CRKP colonization and infection, as well as for increased mortality once infection occurs [3,4,5].

*Klebsiella pneumoniae* (*K. pneumoniae*) is a major cause of hospital-acquired infections, including ventilator-associated pneumonia, bloodstream infections, and intra-abdominal infections, with a particularly high burden in critically ill patients [6,7,8,9]. A recent global analysis estimated that infections caused by *K. pneumoniae* were associated with about eight hundred thousand deaths, and that roughly eighty percent of these deaths involved antimicrobial resistance (AMR) [10,11]. CRKP bloodstream infections carry mortality rates of fifty to sixty percent, far higher than infections caused by susceptible strains [12,13,14,15].

CRKP is now placed in the highest risk tier of the World Health Organization (WHO) priority pathogen lists because of its rapid international spread, high mortality, and limited treatment options [16,17,18,19]. In many surveillance programs, especially in ICUs and other high-dependency areas, it is the most frequently detected carbapenem-resistant *Enterobacterales* species [18,20,21,22].

Carbapenem resistance in *K. pneumoniae* cannot be viewed as a feature of isolated strains. The organism commonly colonizes the human gut and airway without symptoms and serves as a reservoir for later invasive infection [2,23,24,25]. Colonization with CRKP is a significant risk factor for subsequent infection, and many ICU infections arise from strains previously carried in the gastrointestinal tract [5,26,27,28,29]. The gut microbiota plays a central role in this process. Antibiotic exposure and critical illness can disrupt the normal microbial community and reduce colonization resistance, making it easier for carbapenem-resistant *K. pneumoniae* to expand and exchange resistance genes [29,30].

Together, these observations illustrate that CRKP exists at the intersection of antibiotic pressure, intestinal colonization, hospital transmission, and limited therapeutic options. It serves as a model organism for understanding carbapenem resistance as a problem of the broader clinical and microbial system rather than a single resistant clone [18,19,31,32].

Although CRKP has been widely studied, most reviews focus on resistance mechanisms, virulence factors, or molecular epidemiology as separate subjects [33,34,35]. Far less attention has been given to how mobile genetic elements, outer membrane changes, and carbapenemase production act together to shape resistance and affect diagnostic results. This review addresses this gap by bringing these elements together to provide an integrated perspective relevant to molecular surveillance and precision diagnostics.

The aim of this review is to provide an integrative overview of the molecular basis of CRKP, with emphasis on mobile genetic elements that carry carbapenemase genes and on how these determinants interact with hospital environments and the gut microbiota. A second goal is to connect these molecular insights to current and emerging precision diagnostic approaches, including nucleic acid amplification tests, loop-mediated isothermal amplification, and CRISPR-based assays, and to show how improved detection can support infection control and antimicrobial stewardship in high-risk settings [21,28,36].

**Figure 1:**
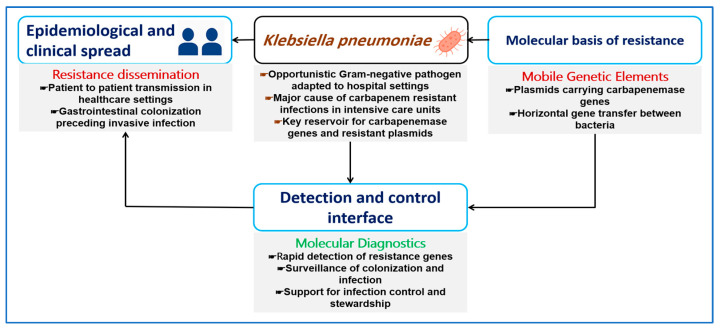
Conceptual overview of CRKP illustrating the links between molecular resistance mechanisms, clinical dissemination, and diagnostic control. Mobile genetic elements drive the spread of carbapenemase genes and define key targets for molecular detection. In healthcare settings, particularly intensive care units, gastrointestinal colonization often precedes invasive infection. Molecular diagnostics support early detection, surveillance, and infection control to limit transmission and guide antimicrobial stewardship.

Figure 1 summarizes the central role of CRKP across molecular resistance mechanisms, clinical spread in healthcare settings, and diagnostic control. It illustrates how mobile genetic elements drive carbapenem resistance, how resistant strains disseminate among patients, and how molecular diagnostics support early detection and containment.

## 2. Carbapenem Resistance as a Genetic System in *K. pneumoniae*

### 2.1. Vertical and Horizontal Inheritance of Resistance

Carbapenem resistance in *K. pneumoniae* spreads through both vertical and horizontal processes. Vertical inheritance occurs when a successful resistant clone expands within a hospital or across a wider region and passes its resistance determinants directly to daughter cells. Whole genome sequencing studies show that a limited number of high-risk lineages, including ST258, ST512, ST11, and ST307, account for many CRKP isolates worldwide, reflecting the strong impact of clonal expansion [37,38].

Horizontal gene transfer occurs alongside clonal spread and allows resistance genes to move between unrelated strains and even different species. In *K. pneumoniae*, carbapenemase genes such as *blaKPC* and *blaNDM* are often carried on conjugative plasmids and other mobile genetic elements that transfer readily between *Enterobacterales* in the gut or in hospital environments [39,40,41]. As a result, a successful clone can acquire new resistance genes, and susceptible strains can become carbapenem resistant without any new introduction from outside the hospital. In practice, vertical and horizontal routes operate together, with plasmids moving within and between dominant clones during outbreaks [40,42].

### 2.2. Evolutionary Stability of Resistance Determinants

The long-term success of carbapenem-resistant *K. pneumoniae* depends on the stability of resistance determinants when antibiotic pressure changes. Studies of carbapenemase-encoding plasmids show that some plasmids remain stable during serial passages without antibiotics, with little loss of resistance and minimal changes in plasmid copy number [43,44]. In epidemic lineages that carry plasmid *blaKPC*, experimental work shows that plasmids such as pKpQIL can persist for many generations even without carbapenem exposure, suggesting close adaptation between the plasmid and the host chromosome [45].

Broader evolutionary studies indicate that plasmid-borne resistance can remain stable when plasmid carriage has a low fitness cost, when plasmids transfer efficiently between hosts, or when compensatory evolution reduces the initial burden of resistance [46,47,48]. For CRKP, this means that once a plasmid adapts to *K. pneumoniae* and related *Enterobacterales* in the gut, it may persist for long periods even if carbapenem use declines. This helps explain why reversing carbapenem resistance is difficult once it becomes established [40,48].

### 2.3. Fitness Costs and Compensatory Mechanisms

Acquisition of carbapenemase genes or other resistance determinants often carries an initial fitness cost. In vitro studies of *K. pneumoniae* and related species show that plasmids carrying *blaNDM* or other resistance genes can slow growth, reduce competitiveness in mixed cultures, or decrease virulence in infection models [49,50]. These effects would be expected to limit the spread of resistant strains when antibiotic pressure is removed.

However, several lines of evidence show that *K. pneumoniae* can rapidly overcome these costs. Experimental evolution studies with New Delhi metallo-beta-lactamase plasmids demonstrate that adaptive mutations in either the plasmid or the chromosome can reduce the fitness burden and allow stable plasmid maintenance [49,51]. Transcriptomic studies of epidemic *K. pneumoniae* carrying pKpQIL indicate that global shifts in gene expression may further rebalance cellular metabolism after plasmid acquisition, reducing fitness penalties [45].

Clinical and genomic studies support these findings. In outbreak lineages such as ST258 and ST11, carbapenemase plasmids often show little measurable impact on growth in standard media yet remain highly stable and transmissible, consistent with compensatory evolution during their spread [37,38,40]. Within-host studies also show that resistant *K. pneumoniae* can accumulate additional mutations over time that fine-tune the balance between resistance, virulence, and fitness under the complex conditions of the human body [52,53].

**Figure 2:**
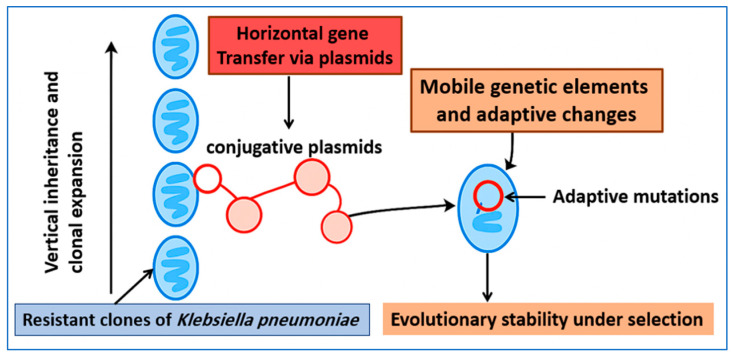
Conceptual model of carbapenem resistance in *K. pneumoniae* as an integrated genetic system. Resistance is maintained through vertical inheritance during clonal expansion and through horizontal gene transfer mediated by conjugative plasmids carrying carbapenemase genes. Following plasmid acquisition, adaptive mutations can reduce fitness costs and promote the long-term stability of resistant lineages under selective pressure.

Figure 2 illustrates how carbapenem resistance in *K. pneumoniae* emerges from the combined effects of clonal expansion, plasmid-mediated gene transfer, and adaptive changes that stabilize resistance across clinical and ecological settings.

As a result, carbapenem resistance in *K. pneumoniae* is best understood as a dynamic genetic system rather than a fixed property of individual strains. Resistance is maintained through the combined effects of clonal expansion and frequent horizontal gene transfer, while the evolutionary stability of carbapenemase-carrying plasmids supports long-term persistence even in the absence of strong antibiotic pressure. The ability of *K. pneumoniae* to compensate for the fitness costs associated with resistance further explains why resistant lineages often persist despite improved antibiotic stewardship and infection control.

These features show that carbapenem resistance in *K. pneumoniae* develops through the interaction of genetic mobility, evolutionary adaptation, and clinical selection. This perspective provides the foundation for Section 3, which examines the mobile genetic elements that carry carbapenemase genes and the molecular architecture that supports their successful dissemination.

## 3. Mobile Genetic Elements Driving Carbapenem Resistance

Carbapenem resistance in *K. pneumoniae* operates as a dynamic genetic system shaped by clonal expansion, horizontal gene transfer, and adaptive change. This section focuses on the mobile genetic elements that carry and disseminate carbapenemase genes.

### 3.1. Plasmid Incompatibility Groups and Epidemic Backbones

In CRKP, carbapenemase genes are most often carried on conjugative plasmids that transfer readily between strains and across species. Population studies show that a limited number of epidemic plasmid backbones, sometimes referred to as epidemic resistance plasmids, account for much of this spread in hospitals. These plasmids are usually large, self-transmissible, and stably maintained during cell division, which allows them to persist once they enter a successful clone [40,54].

Plasmids carrying *blaKPC* have been reported in several incompatibility groups, including IncFII, IncFIIK, IncN, IncL/M, IncA/C, IncR, IncX, and IncHI. Among these, IncFIIK and related IncF plasmids are especially common in the global *K. pneumoniae* ST258 lineage and related clones, and played a central role in the early international spread of *blaKPC* [40,55,56]. Plasmids in the IncN and IncL/M groups have also been linked repeatedly to *blaKPC* and *blaOXA-48-like* dissemination in clinical settings, highlighting the contribution of diverse plasmid backbones to the CRKP problem [57,58].

The pKpQIL family of IncFIIK plasmids is a well-characterized example. These plasmids carry *blaKPC* within a defined transposon structure and encode a full conjugation system as well as stability and maintenance functions. Genomic studies show that pKpQIL-like plasmids have undergone structural remodeling during their spread. Despite this remodeling, the core backbone that supports conjugation and stable replication remains conserved, underscoring the importance of backbone architecture for long-term success [55,59,60].

Carbapenemase genes other than *blaKPC* also rely on characteristic plasmid platforms. *blaNDM* is found on a range of plasmids, including IncF, IncX3, IncHI, and IncC, which tend to be more heterogeneous and recombination prone than classical *blaKPC* plasmids [61,62,63]. Recent reports describe IncHI5 or IncHI5-like plasmids co-carrying *blaNDM* with other resistance determinants in CRKP, illustrating how broad-host-range plasmids can accumulate multiple last-line resistance genes within a single transferable element [62,64].

These data show that carbapenem resistance in *K. pneumoniae* is closely tied to a limited set of successful plasmid backbones. These backbones provide the replication, maintenance, and conjugation functions that allow carbapenemase genes to persist in high-risk lineages and cross species boundaries in hospital environments [40,56].

### 3.2. Transposons, Integrons, and Insertion Sequences Surrounding Carbapenemase Genes

Plasmids alone do not account for the fine-scale mobility of carbapenemase genes. Transposons, integrons, and insertion sequences act as smaller mobile units that capture these genes and move them between plasmid and chromosomal contexts. For *blaKPC*, the composite transposon Tn4401 is the best-known example. Tn4401 carries *blaKPC* flanked by insertion sequences such as ISKpn6 and ISKpn7 and can insert into many plasmids and genomic regions. This mobility has enabled *blaKPC* to spread across diverse plasmid backbones and host species [55,58,59].

For *blaNDM* and *blaVIM*, class 1 integrons and Tn3 family transposons are frequent genetic contexts. These structures often place the carbapenemase gene within a cassette array that may include determinants for other antibiotic classes. They are often bordered by insertion sequences such as IS26, which promote recombination and rearrangement [58,61,65]. Because of this ongoing reshuffling, the same carbapenemase gene can appear in multiple genetic environments within a single outbreak [63].

Insertion sequences can also drive resistance evolution on their own. IS26 and related elements can mediate deletions, inversions, and duplications, and they can bring together separate resistance regions into larger composite structures. Studies of CRKP and related *Enterobacterales* show that clusters of insertion sequences flank many resistance islands and may support both the capture of new genes and the movement of existing resistance modules between plasmids [65,66,67].

Overall, these smaller mobile elements serve as the fine wiring of the resistance network. They allow carbapenemase genes to integrate into different plasmid and chromosomal backgrounds, move between backbones with favorable transmission properties, and accumulate with other resistance determinants in complex islands [40,58,61].

### 3.3. Co Selection and Persistence Mechanisms

Mobile genetic elements in CRKP rarely carry a single resistance gene. Instead, carbapenemase plasmids often contain determinants for other antibiotic classes, heavy metals, biocides, and virulence-associated traits. This clustering creates strong opportunities for co-selection, in which exposure to one compound indirectly maintains resistance to others [40,68].

Environmental and clinical studies provide clear examples. Plasmids from *K. pneumoniae* and related species recovered from hospital wastewater and marine environments often co-locate carbapenemase genes with metal resistance operons, such as mercury resistance (*mer*) clusters, and with genes for additional multidrug efflux systems [69,70,71]. In these settings, heavy metals and other pollutants can maintain carbapenemase plasmids even when carbapenem use is limited, because plasmid-carrying cells gain a survival advantage under non-antibiotic stress [69,72].

Beyond hospital settings, carbapenem resistance in *K. pneumoniae* should be viewed within a One Health framework. Genomic studies show that broad host range plasmids, including IncHI2 and related backbones, circulate across human, animal, and environmental reservoirs. These plasmids contribute to the spread of clinically relevant resistance genes [34,73]. Similar resistance plasmids have been identified in livestock, companion animals, wastewater, and human clinical isolates. This pattern highlights the potential for interspecies transmission and long-term environmental persistence of carbapenem resistance determinants [73,74].

Additional persistence mechanisms are encoded directly on plasmid backbones. Many epidemic resistance plasmids in CRKP encode toxin-antitoxin systems and partitioning modules that stabilize inheritance during cell division and reduce plasmid loss in the absence of antibiotics [40,54]. Some plasmids also carry colonization and virulence factors, including siderophore systems and capsule regulators, which may improve bacterial fitness in the host and further support plasmid retention in successful lineages [75,76].

These findings show that carbapenem resistance in *K. pneumoniae* is sustained by mobile genetic elements operating at several levels. A limited set of epidemic plasmid backbones provides stability and efficient transfer, while transposons and insertion sequences enable carbapenemase genes to move between genetic contexts and accumulate with other resistance traits. Co-selection by non-carbapenem agents and plasmid-encoded stability systems enhances persistence even when carbapenem use is reduced.

**Figure 3:**
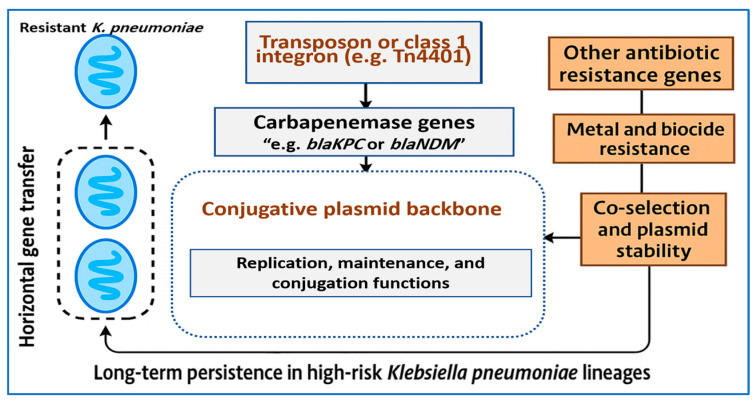
Schematic overview of the mobile genetic architecture supporting carbapenem resistance in *K. pneumoniae*. Carbapenemase genes are captured by transposons or class 1 integrons and integrated into conjugative plasmid backbones that provide replication, maintenance, and transfer functions. These plasmids often carry additional resistance and stability determinants, enabling co-selection under different selective pressures and promoting long-term persistence in high-risk *K. pneumoniae* lineages.

This integrated view of mobile genetic elements sets the stage for the next section, which examines the molecular determinants of carbapenem resistance and the pathways through which these mechanisms shape clinical phenotypes. Figure 3 shows how carbapenem resistance in *K. pneumoniae* is maintained by conjugative plasmids that integrate carbapenemase genes with broad resistance and stability modules, enabling persistence and spread under selective clinical pressures.

### 3.4. Convergence of Hypervirulence and Carbapenem Resistance (hv-CRKP)

Recent genomic and epidemiological studies have documented the emergence of *K. pneumoniae* strains that combine carbapenem resistance with hypervirulence, commonly referred to as hypervirulent carbapenem-resistant *K. pneumoniae* (hv-CRKP), and these strains have been increasingly reported from hospital settings worldwide [35,77,78]. This convergence represents a major evolutionary shift for *K. pneumoniae* and is associated with increased disease severity, limited treatment options, and higher mortality rates [77,79].

At the molecular level, hv-CRKP most commonly arises through the acquisition of large virulence plasmids by classical carbapenem-resistant lineages, rather than through the gradual accumulation of individual virulence traits [35,80]. These virulence plasmids are typically related to the pLVPK family and encode key determinants such as rmpA and rmpA2, which enhance capsule production, as well as siderophore biosynthesis systems including aerobactin and salmochelin that promote iron acquisition and invasive potential [79,81].

Genomic analyses have shown that pLVPK-like virulence plasmids have increasingly been acquired by high-risk carbapenem-resistant clones, particularly sequence type ST11 and, less frequently, ST258, leading to the emergence of hv-CRKP in endemic and epidemic settings [77,78]. The incorporation of these plasmids into multidrug-resistant backgrounds results in strains that retain resistance to last-line antibiotics while gaining enhanced virulence, and this convergence is primarily mediated by horizontal gene transfer under strong selective pressure in healthcare environments [35,79].

The emergence of hv-CRKP highlights the need for molecular surveillance strategies that simultaneously assess resistance and virulence determinants, as screening based solely on carbapenemase genes fails to capture the full clinical risk posed by these convergent strains [35,82].

## 4. Molecular Determinants of Carbapenem Resistance in *K. pneumoniae*

The mobile genetic elements described in Section 3 carry resistance determinants that act at the cell envelope and in the periplasm. In *K. pneumoniae*, carbapenem resistance usually results from a combination of carbapenemase production, reduced outer membrane permeability, and, in some strains, increased efflux. These mechanisms work together to lower the concentration of active drug at penicillin-binding proteins and protect the cell from β-lactam killing [33,83].

### 4.1. Carbapenemase Enzymes

Carbapenemases are β-lactamases that hydrolyze carbapenems and many other β-lactams. In *K. pneumoniae*, the main families include class A enzymes such as *blaKPC*, class B metallo-β-lactamases such as *blaNDM*, *blaVIM*, and *blaIMP*, and class D OXA-48-like enzymes encoded by *blaOXA-48* and related genes [33,40,84]. These genes are usually carried on conjugative plasmids and are often linked with additional resistance determinants, which supports rapid spread in clinical settings.

Class A *K. pneumoniae* carbapenemase (KPC) enzymes are now among the most common carbapenemases in *K. pneumoniae*. They are serine β-lactamases with broad activity against penicillins, cephalosporins, and carbapenems [33,85]. Early international spread was driven by high-risk clones such as ST258 that carried *blaKPC* on pKpQIL-like IncFIIK plasmids, which combine efficient conjugation with stable maintenance systems [60,86]. Many KPC variants have since been described, including forms with reduced susceptibility to β-lactamase inhibitors, which complicates treatment and diagnostic interpretation [87,88].

Metallo-β-lactamases such as NDM, VIM, and IMP use zinc ions at the active site and inactivate almost all β-lactams except monobactams [33,89]. In *K. pneumoniae*, *blaNDM* has been detected on several broad-host-range plasmids, including IncF, IncX3, IncHI, and IncC, and often coexists with additional resistance genes on complex plasmid backbones [40]. Strains that carry *blaNDM* or other metallo-β-lactamases usually show resistance to most β-lactams and retain few therapeutic options.

OXA-48-like enzymes form a distinct group of class D carbapenemases. They hydrolyze carbapenems less efficiently than KPC or NDM but often occur with extended-spectrum β-lactamases or porin loss, which together yield high-level resistance [33,90,91]. *blaOXA-48* is most often found on IncL/M plasmids with strong epidemic potential and is now common in *K. pneumoniae* across many regions [85,92,93]. Co-carriage of *blaOXA-48* with *blaNDM* or *blaKPC* in the same isolate is increasingly reported and produces an extreme resistance phenotype [94,95,96].

Overall, carbapenemase production is the dominant molecular driver of carbapenem resistance in *K. pneumoniae*, but it rarely acts alone. Resistance levels and clinical phenotypes depend strongly on the permeability and efflux background described in the following subsections [33,83].

### 4.2. Porin Alterations and Reduced Outer Membrane Permeability

Carbapenems reach their targets in the periplasm by passing through nonspecific outer membrane porins. In *K. pneumoniae*, OmpK35 and OmpK36 are the principal porins involved in this process [97,98]. Many carbapenem-resistant isolates show reduced expression, structural modification, or complete loss of one or both porins. These changes decrease drug influx and raise carbapenem minimum inhibitory concentrations (MICs).

Clinical studies show that loss of both OmpK35 and OmpK36 can convert an extended-spectrum β-lactamase or AmpC-producing strain from carbapenem susceptible to carbapenem resistant [97,99,100]. In several series, non-carbapenemase-producing *K. pneumoniae* with high carbapenem MICs carried ESBL or AmpC enzymes together with inactivating mutations or insertion sequences in *ompK35* and *ompK36* [99,101,102]. Among these two porins, OmpK36 appears especially important for carbapenem entry. In addition to complete porin loss, specific amino acid insertions in the loop 3 region of OmpK36, including glycine aspartate or threonine aspartate duplications, narrow the channel and limit carbapenem entry. These structural changes increase ertapenem and meropenem MICs while still allowing sufficient nutrient uptake to preserve bacterial fitness [103]. Loss of this porin, or narrowing of its channel, has been linked to large increases in ertapenem and meropenem MICs, particularly in high-risk clones such as ST258 [97,100,104].

Porin alterations also affect resistance in carbapenemase-producing strains. Increased copy number of *blaOXA-48* combined with loss of OmpK36 has been associated with higher carbapenem MICs and treatment failure, showing that enzyme levels and outer membrane permeability act together [33,105]. Similar interactions have been reported for KPC- and NDM-producing isolates, where loss of OmpK35 or OmpK36 amplifies the effect of the carbapenemase [40,106,107].

### 4.3. Efflux Pumps and Layered Resistance

Active efflux adds another layer to carbapenem resistance in *K. pneumoniae*. The best studied pump is AcrAB-TolC, a resistance-nodulation-division efflux system that spans the inner membrane, the periplasm, and the outer membrane channel. Experimental work shows that AcrAB contributes to multidrug resistance and can decrease susceptibility to several β-lactams, including carbapenems, especially when overexpressed [108,109,110,111]. Additional pumps such as OqxAB and MdtK have also been associated with higher resistance levels in clinical isolates [112,113,114].

Efflux pumps alone rarely raise carbapenem MICs into the fully resistant range. Their main role is to act together with carbapenemases and porin changes. When outer membrane permeability is reduced and a carbapenemase is present, increased efflux can further lower periplasmic drug concentrations and strengthen clinical resistance [83,98,115].

**Figure 4:**
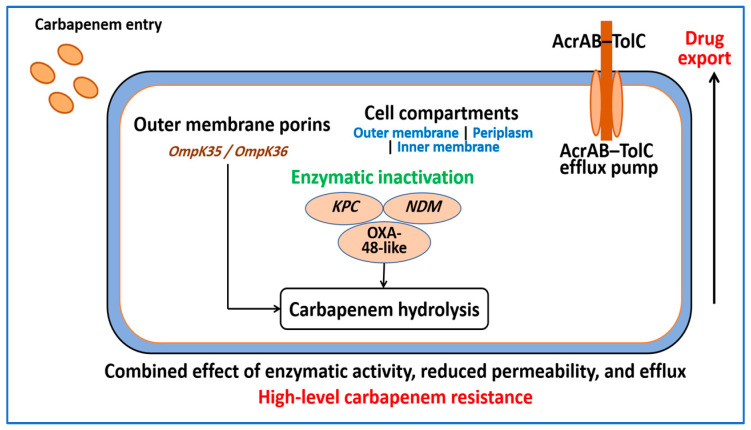
Integrated molecular mechanisms underlying carbapenem resistance in *Klebsiella pneumoniae*. Carbapenem entry through the outer membrane is reduced when porins such as OmpK35 and OmpK36 are altered or expressed at low levels. In the periplasm, carbapenemase enzymes including KPC, NDM, and OXA-48-like hydrolyze carbapenems, and active efflux mediated by the AcrAB–TolC system further decreases intracellular drug levels. The combined effects of enzymatic inactivation, reduced permeability, and efflux produce high-level carbapenem resistance.

Overall, the molecular determinants described in this section show that carbapenem resistance in *K. pneumoniae* does not arise from a single mutation or gene. Instead, it reflects a layered architecture of enzymes, porins, and efflux systems that interact with the plasmid platforms and evolutionary processes outlined in Section 2 and Section 3. This integrated view is essential for interpreting diagnostic results and for designing molecular tests that capture the most relevant resistance pathways. Figure 4 illustrates how carbapenem resistance in *K. pneumoniae* emerges from the combined effects of carbapenemase activity, reduced outer membrane permeability, and active efflux, which together limit the effective drug concentration at bacterial targets.

## 5. The Resistome Beyond the Pathogen: Gut Microbiota and Silent Dissemination

The human gut is a major ecological niche for *K. pneumoniae* and for many other *Enterobacterales* that carry resistance genes. Colonization at this site often precedes invasive disease, and in many patients the infecting strain is identical or closely related to the earlier colonizing strain [23,24,116]. At the same time, the gut microbiota functions as a reservoir of antimicrobial resistance genes and as a platform for horizontal gene transfer [117,118]. This section examines how intestinal colonization, microbiota-mediated gene exchange, and antibiotic-driven dysbiosis together support silent dissemination of carbapenem-resistant *K. pneumoniae*.

### 5.1. Intestinal Colonization and Colonization Resistance Reservoirs

CRKP frequently colonizes the gastrointestinal tract of hospitalized patients, particularly in intensive care units. Rectal carriage of CRKP has been identified as an independent risk factor for subsequent infection and for death in several cohorts [23,119,120]. In many cases, isolates from bloodstream infections or pneumonia match the earlier gut colonizing strain, confirming the intestine as a primary reservoir for later invasive disease [116,121].

Despite this risk, the native gut community normally provides colonization resistance. A diverse microbiota limits the expansion of exogenous *Enterobacterales* through competition for nutrients, production of inhibitory metabolites, and stimulation of mucosal immunity [118,122,123]. Clinical and experimental studies show that loss of key commensals is associated with overgrowth of multidrug-resistant organisms, including carbapenem-resistant *Enterobacterales* [30,124,125]. In this way, the gut microbiota functions as a dynamic resistance reservoir. When intact, it can buffer colonization; when disturbed, it can allow resistant strains such as CRKP to expand silently to high densities.

Occult colonization adds further complexity. Animal models and human surveillance studies indicate that carbapenem-resistant *Enterobacterales* may persist at very low levels that escape routine screening but can later expand after antibiotic exposure or critical illness [126,127]. These silent carriers contribute to hospital transmission and complicate infection control.

### 5.2. Microbiota Mediated Horizontal Gene Transfer in the Gut

The gut microbiota contains a dense network of bacteria that exchange genetic material at high frequency. Metagenomic surveys show that commensal gut species harbor large collections of resistance genes and that horizontal gene transfer events occur more often in the intestine than in many other environments [117,128]. Conjugative plasmids, integrative elements, and bacteriophages all participate in this exchange, with conjugation regarded as the dominant route for resistance gene transfer between *Enterobacterales* in the human gut [129,130].

Carbapenemase genes such as *blaKPC* and *blaNDM* are often carried on plasmids that can move between commensal species and *K. pneumoniae* during intestinal colonization [131,132,133]. Studies combining stool culturing with sequencing have documented shared plasmids and resistance gene cassettes in different *Enterobacterales* from the same patient, supporting active in vivo transfer [124,134]. In this context, the gut serves not only as a reservoir of CRKP but also as a site where new resistance combinations can emerge before they appear in clinical isolates.

This microbiota-level resistome blurs the line between pathogen and commensal. Organisms that are harmless in most settings can carry plasmids or transposons that later move into *K. pneumoniae* and other high-risk pathogens [117,135]. Understanding these within-host exchange pathways is therefore essential for any strategy that aims to interrupt the emergence of new carbapenem-resistant clones.

### 5.3. Antibiotic-Driven Dysbiosis and Silent Dissemination of CRKP

Antibiotic therapy is one of the main forces that reshapes the gut community in hospitalized patients. Broad spectrum regimens reduce microbial diversity, deplete anaerobic commensals, and alter short chain fatty acid production, all of which weaken colonization resistance [136,137,138]. Observational studies show that these antibiotic-induced changes are associated with higher carriage rates of multidrug-resistant *Enterobacterales*, longer persistence of colonization, and increased risk of later infection [127,139,140]. Under these conditions, carbapenemase plasmids can be maintained without direct carbapenem exposure through co-selection by other antibiotics and by non-antibiotic pressures such as inflammation or nutritional stress.

Clinically, this means that patients who receive repeated or prolonged antibiotic courses may carry CRKP and related organisms for months after treatment ends, often without symptoms [122,125,127]. These silent carriers act as a hidden reservoir for hospital transmission and as a starting point for invasive infections when new risk factors, such as intensive care admission or invasive procedures, arise.

**Figure 5:**
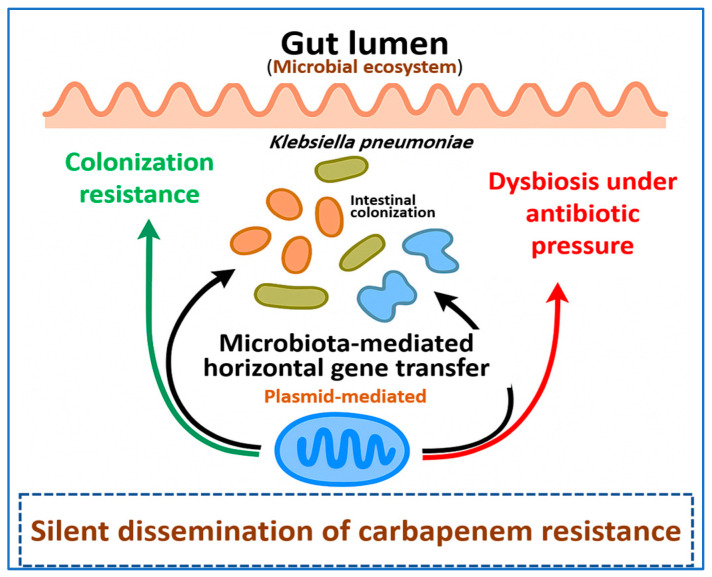
Schematic representation of the gut microbiota as a reservoir for carbapenem resistance beyond overt infection. In an intact gut ecosystem, a diverse microbial community provides colonization resistance that limits expansion of *Klebsiella pneumoniae*. Antibiotic exposure disrupts this balance and leads to dysbiosis, promoting intestinal colonization by *K. pneumoniae* and enabling microbiota-mediated horizontal gene transfer. These processes allow carbapenem resistance determinants to circulate and persist silently within the gut, creating a hidden reservoir that supports later transmission and infection.

Figure 5 illustrates how antibiotic-driven disruption of the gut microbiota weakens colonization resistance and promotes silent exchange of resistance determinants between *K. pneumoniae* and resident bacteria, supporting persistence and dissemination of carbapenem-resistant strains.

## 6. Molecular Diagnostics for CRKP

Rapid and reliable detection of carbapenemase genes is now central to the management of CRKP in both clinical care and infection control. Molecular assays allow direct identification of resistance determinants from clinical samples or screening swabs, often within a few hours. Most current platforms target the major carbapenemase families found in *K. pneumoniae*, particularly *blaKPC*, *blaNDM*, *blaOXA-48-like*, *blaVIM*, and *blaIMP* [141].

### 6.1. Nucleic Acid Amplification Tests for Carbapenemase Genes

Conventional and real-time polymerase chain reaction (PCR) assays were the first molecular tools developed for carbapenemase detection in *K. pneumoniae*. Early multiplex real-time PCR protocols could detect *blaKPC*, *blaNDM*, and *blaOXA-48-like* directly from rectal swabs or cultured isolates within a few hours, with high analytical sensitivity [142,143]. These in-house tests remain widely used in reference laboratories because they are flexible and can be updated when new alleles emerge.

Commercial nucleic acid amplification tests (NAATs) apply the same principles within closed, standardized systems. Cartridge-based platforms such as Xpert Carba-R detect the main carbapenemase families from rectal swabs or positive blood cultures with a turnaround time of about one hour [144,145,146]. Meta-analyses and cohort studies show very high sensitivity and specificity for *blaKPC* and good performance for *blaNDM*, *blaOXA-48-like*, *blaVIM*, and *blaIMP* [147,148]. These assays are therefore widely used for screening high-risk patients and confirming carbapenemase production.

Despite these strengths, NAATs have clear limitations. They detect only the targets included in the assay panel and may miss rare carbapenemase families or new variants [141]. They report gene presence rather than gene expression, and they do not detect additional mechanisms such as porin loss or efflux pump upregulation. They also require specific instruments, a steady supply of cartridges, and trained personnel, which can limit their use in low-resource settings.

### 6.2. Loop-Mediated Isothermal Amplification Assays

Loop-mediated isothermal amplification (LAMP) offers an alternative that works at a single reaction temperature and does not require a thermocycler. LAMP assays targeting *K. pneumoniae* and common carbapenemase genes can detect low numbers of bacteria within 30–60 min, often with simple visual readouts such as color change or lateral flow strips [149,150,151]. Evaluations of LAMP for *blaKPC*, *blaNDM*, and other carbapenemase genes report analytical sensitivities that match or exceed standard PCR and show good agreement with reference methods when applied to clinical isolates or rectal swabs [152,153,154].

Because LAMP operates at a constant temperature and can be read with basic devices, it is attractive for decentralized settings, including smaller hospitals or laboratories with limited infrastructure. It can also be integrated into low-cost DNA chromatography or dipstick formats to detect several carbapenemase families in parallel [150,151].

However, LAMP presents its own challenges. Primer design is complex, and non-specific amplification can occur if reactions are not carefully optimized. The high efficiency of amplification increases the risk of carry-over contamination if workflows are not well separated. Multiplexing beyond a few targets remains difficult, which can limit coverage in regions where several carbapenemase families co-circulate.

### 6.3. CRISPR-Based Diagnostic Platforms

New diagnostic platforms now use clustered regularly interspaced short palindromic repeats (CRISPR) and associated Cas enzymes to detect carbapenemase genes with very high sensitivity. These assays couple an isothermal pre-amplification step, such as recombinase polymerase amplification, with a CRISPR–Cas12a or Cas13a reaction that cleaves a reporter molecule when the target sequence is present [155,156].

Prototype assays have been developed that detect *blaKPC* alone or panels of five major carbapenemase genes in a single reaction [157,158]. These systems reach very low detection limits, sometimes down to a few copies of DNA per microliter, and provide fluorescence or lateral-flow readouts in less than one hour. Early evaluations indicate that these tools can distinguish closely related carbapenemase alleles and may be adapted for portable, near-patient testing.

At present, most CRISPR-based assays remain in the research or early translational phase. They require careful optimization of both the amplification step and the CRISPR components, and routine diagnostic experience is still limited. Standardization, regulatory approval, and cost-effectiveness studies are needed before wider clinical adoption.

### 6.4. Capabilities and Current Limitations

Over the past decade, molecular assays have reshaped CRKP detection by allowing rapid identification of key carbapenemase genes from clinical isolates and surveillance samples [159]. NAATs, including multiplex PCR and related real-time platforms, now provide standardized detection of *blaKPC*, *blaNDM*, *blaOXA-48-like*, and other major carbapenemase genes in routine practice [160,161].

LAMP-based assays extend this capability to laboratories with limited infrastructure because they operate at a single temperature and can be paired with simple visual or portable readouts while still detecting the main carbapenemase families in *Enterobacterales* [151,152,162]. More recently, CRISPR-guided platforms using Cas12 or Cas13 enzymes with PCR, recombinase-based amplification, or LAMP have achieved very high analytical sensitivity for *blaKPC*, *blaNDM*, and related targets, with assays developed specifically for CRKP detection [163,164,165].

Despite these advances, current molecular tests focus mainly on known resistance genes and selected alleles. They therefore may miss non-carbapenemase mechanisms such as combinations of extended-spectrum β-lactamases with porin loss or efflux, and they may fail to detect novel or uncommon carbapenemase variants outside the assay panel [166,167,168]. These blind spots help explain ongoing discrepancies between genotypic and phenotypic results and highlight the need to complement rapid molecular methods with broader phenotypic testing or sequencing in critical settings [159,167].

Molecular assays enable rapid detection of carbapenemase genes, but they do not assess how bacteria respond to antibiotic exposure. Recent phenotypic approaches address this gap by measuring bacterial activity after short drug challenge. Wu et al. showed that changes in intracellular enzyme activity can be used to rapidly determine antimicrobial susceptibility and estimate MICs for antibiotics with different mechanisms of action [169]. Using a digital enzyme-based approach, Wu et al. further demonstrated that single-cell metabolic activity can be quantified to assess bacterial viability and phenotypic drug response without relying on predefined resistance genes [170]. These methods reflect the combined effects of resistance mechanisms, including enzyme production, reduced drug entry, and metabolic stress.

In addition to metabolic assays, whole-cell bacterial biosensors offer another phenotypic strategy for resistance detection. Jeffs et al. developed a luminescent biosensor that produces a measurable signal in response to carbapenemase activity, enabling rapid identification of carbapenemase-producing Enterobacterales [171]. Because the signal depends on enzyme function rather than gene presence alone, this approach can detect active resistance even when gene expression is low or genetic variants fall outside routine molecular panels.

Overall, enzyme-based susceptibility testing and bacterial biosensors complement genotypic diagnostics by providing functional confirmation of resistance. These approaches reduce discrepancies between genetic and phenotypic results and improve the clinical interpretation of rapid molecular tests, particularly in high-risk hospital settings.

The clinical impact of these gaps, and their implications for patient management and infection control, are addressed in the next section on diagnostic blind spots and emerging challenges. The application of these molecular diagnostic approaches in different hospital contexts is summarized in Table 1.

## 7. Diagnostic Blind Spots and Emerging Challenges

### 7.1. Silent Carriers and Low-Expression Carbapenemase Genes

Molecular screening has improved the detection of patients who carry carbapenemase-producing *K. pneumoniae*, but it does not identify every carrier. Rectal screening studies show that the sensitivity of culture and nucleic acid amplification tests depends strongly on bacterial load and on how samples are collected and processed. In several cohorts, some colonized patients were detected only after broth enrichment or only by polymerase chain reaction, while direct culture on selective plates remained negative [176,177].

These findings suggest that patients with low-level colonization may remain undetected during routine screening and act as silent carriers in high-risk wards. During one outbreak of carbapenemase-producing *K. pneumoniae*, real-time polymerase chain reaction on rectal swabs identified more carriers than selective culture, even though both methods analyzed the same samples [178,179].

Expression level also affects detection. Phenotypic assays that rely on enzyme activity, including some colorimetric carbapenemase tests, are less sensitive when carbapenemase production is low or when there has been limited carbapenem exposure before sampling. Reviews of screening approaches for intestinal carriage of carbapenemase-producing *Enterobacterales* show that performance varies between centers and that sensitivity often declines when bacterial loads are close to the limit of detection [176,180].

These data indicate that negative screening results cannot fully exclude colonization, especially in patients who recently received antibiotics, have intermittent shedding, or carry very low numbers of organisms in the gut. As a result, silent carriers remain an important diagnostic blind spot and may contribute to ongoing transmission despite apparently adequate screening programs [181].

### 7.2. Undetected Plasmid and Environmental Reservoirs

Routine diagnostics focus primarily on patient samples, yet the genetic material that drives carbapenem resistance often persists outside obvious clinical isolates. Genomic surveys of hospital plumbing and sink drains have revealed dense biofilms containing *Enterobacterales* with carbapenemase plasmids, including elements closely related to those from patients on the same wards [182,183].

In several hospitals, carbapenemase-producing *Enterobacterales* have been recovered from sinks and drains even when no colonized patient was identified nearby, suggesting either unrecognized carriers or long-term environmental persistence. In some investigations, strains and plasmids from sink drains matched clinical isolates by whole genome sequencing, supporting the idea of a shared reservoir that is not routinely sampled [184,185].

Public health agencies now recognize sinks, drains, showers, and toilets as potential hidden reservoirs of carbapenem-resistant *Enterobacterales*. Their guidance recommends targeted environmental cultures when transmission persists despite standard control measures [186].

Routine diagnostic workflows rarely include systematic environmental sampling or screening of staff and visitors, and almost never track plasmids independently of their bacterial hosts. As a result, clinically important carbapenemase plasmids may continue to circulate in plumbing biofilms, wastewater, and unsampled host populations while patient cultures appear controlled. This gap between what is tested and where resistance genes persist represents a second major diagnostic blind spot [187,188].

### 7.3. Evolution Beyond Current Diagnostic Targets

Most molecular assays used in clinical laboratories target specific carbapenemase genes and common variants, including *blaKPC*, *blaNDM*, *blaVIM*, *blaIMP*, and *blaOXA-48-like*. These panels perform well for the major globally distributed enzymes but do not capture the full diversity of known and emerging carbapenemases. Public health guidance notes that isolates with positive phenotypic carbapenemase tests but negative results in standard gene panels often carry rare variants, uncommon carbapenemase families, or novel enzymes not yet included in commercial kits [189,190].

Evaluations of lateral flow assays and other rapid platforms have also reported occasional false negatives for specific variants, particularly within diverse families such as IMP and some VIM enzymes. Small sequence changes can interfere with probe or antibody binding while still preserving carbapenemase activity [191,192].

At the same time, evolutionary studies of carbapenem-resistant *K. pneumoniae* show that new resistance configurations continue to arise as plasmids recombine, insertion sequences restructure genetic islands, and extended-spectrum β-lactamase producers acquire additional mechanisms that reduce carbapenem susceptibility. Whole genome sequencing can detect many of these events but is not yet available in real time in most hospitals and is typically reserved for selected outbreaks or reference investigations [193,194].

**Figure 6:**
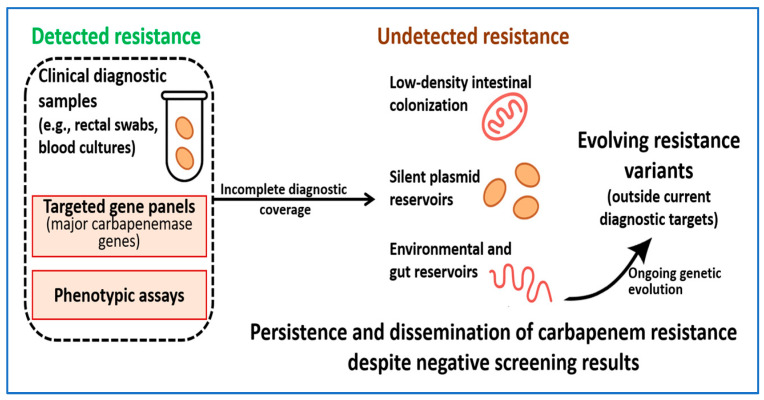
Conceptual overview of diagnostic blind spots that allow carbapenem resistance to persist despite routine screening. Standard diagnostics focus on defined sample types and targeted gene or phenotypic assays and therefore detect many established resistance mechanisms. In contrast, low-density intestinal colonization, silent plasmid reservoirs, environmental and gut niches, and emerging resistance variants outside current diagnostic targets may remain undetected, enabling continued persistence and dissemination.

These dynamics mean that diagnostic targets remain slightly behind the organisms they are designed to identify. Without regular updates to primer sets, probe panels, and interpretation rules, laboratories risk missing unusual carbapenemase genes or novel combinations of resistance determinants that fall outside existing algorithms [189]. Figure 6 summarizes the conceptual basis of these diagnostic blind spots.

### 7.4. Implications for Clinical Care and Surveillance

The blind spots outlined above have direct implications for patient management and infection control. Silent carriers and low-density colonization can lead to unexpected transmission events, especially in intensive care and other high-risk units, even when admission screening results are negative. Environmental reservoirs and unrecognized plasmid pools can also sustain low-level endemicity and may trigger new outbreaks after clinical incidence appears to decline [176,186].

From a diagnostic perspective, negative results from targeted molecular tests must be interpreted with caution when clinical suspicion remains high or when local epidemiology suggests the presence of unusual carbapenemases. Many guidelines now recommend pairing genotypic methods with broad phenotypic assays and, when necessary, whole genome sequencing to resolve discordant findings and monitor emerging resistance mechanisms [180,190].

For surveillance, these challenges support a more integrated strategy that links patient screening, environmental monitoring, and regular reassessment of diagnostic panels. In the context of carbapenem-resistant *K. pneumoniae*, such an approach recognizes that the resistance problem extends beyond individual isolates and reflects a broader and evolving genetic system that current tests capture only in part. This perspective sets the foundation for the next section, which considers how molecular tools may shift from passive detection toward active disruption of resistance trajectories.

## 8. From Detection to Disruption: Can Molecular Tools Alter Resistance Routes?

Molecular diagnostics for CRKP are now widely used in clinical care, yet they remain mainly descriptive. A central question is whether the same molecular logic can be used to weaken or remove resistance determinants. This section outlines three experimental approaches that shift from detecting resistance toward actively disrupting it.

### 8.1. CRISPR-Based Targeting of Resistance Determinants

Native and engineered CRISPR–Cas systems can, in principle, be directed to cut plasmid or chromosomal DNA that carries resistance genes. In *Klebsiella* species, type I and type IV CRISPR–Cas loci occur on chromosomes and plasmids, and spacer content often matches mobile genetic elements, suggesting a role in limiting incoming plasmids [195,196,197]. In some clinical *K. pneumoniae* collections, strains with active CRISPR–Cas systems are less likely to carry *blaKPC* plasmids, which supports this idea at a population level [197,198].

Engineered CRISPR tools extend this concept by directing Cas nucleases against specific resistance loci. In vitro and animal studies show that CRISPR–Cas9 constructs can selectively cleave plasmids carrying resistance genes, remove them from *Enterobacterales*, and restore susceptibility to β-lactams [199,200]. Modeling work also suggests that CRISPR-based clearance, combined with antibiotic treatment, could shift mixed populations toward susceptible cells if enough bacteria receive the CRISPR payload [201].

For CRKP, these findings raise the possibility of directing CRISPR activity against *blaKPC*, *blaNDM*, or associated plasmid backbones. However, major practical barriers remain. Delivery into dense gut communities or infected tissues is still inefficient, escape mutants can emerge, and off-target effects on the microbiota are not fully understood [202,203]. At present, CRISPR-based strategies should be viewed as proof-of-concept tools that illustrate what may be possible, rather than near-term therapies for CRKP infection.

### 8.2. Anti-Plasmid Strategies and Plasmid Curing

Because carbapenem resistance in *K. pneumoniae* is often plasmid mediated, direct anti-plasmid strategies are appealing. Laboratory studies have shown that plasmid incompatibility, interference with plasmid replication, and disruption of toxin and antitoxin systems can remove resistance plasmids from *Enterobacterales* in vitro and in animal models [199,204]. In one gut colonization model, exploiting incompatibility between related plasmids led to stable loss of resistance plasmids and restoration of antibiotic susceptibility [199].

Chemical plasmid-curing agents, including detergents and intercalating compounds, have also been described, but many work only at concentrations unsuitable for human use [203,204]. More selective small molecules that block plasmid replication or stability are under investigation, but none are ready for clinical testing in CRKP [204].

From a systems perspective, plasmid-focused strategies are attractive because they could reduce resistance across multiple species in the gut or environment at once. At the same time, any intervention that alters plasmid ecology on a broad scale requires careful evaluation of potential effects on beneficial plasmids, microbiota composition, and horizontal gene flow.

### 8.3. Phage-Assisted Delivery and Phage Therapy

Bacteriophages naturally deliver DNA into *K. pneumoniae* and other *Enterobacterales*, which places them at the center of two related strategies. The first is classical phage therapy, where lytic phages are used as precision antimicrobials against CRKP. Case reports and early clinical series describe successful treatment of severe CRKP infections, often in combination with antibiotics, and recent work has isolated phages active against high-risk clones such as ST307 and ST147 [205,206,207,208].

The second strategy uses phages as engineered vectors to deliver CRISPR–Cas systems or other anti-plasmid payloads. Experimental phage and CRISPR constructs have been used to remove resistance plasmids in *Escherichia coli* and other *Enterobacterales*, including in mouse gut models, and to kill only those cells that carry a targeted resistance gene [200,202]. In principle, this approach could be adapted to CRKP by packaging guides directed at *blaKPC*, *blaNDM*, or specific plasmid backbones.

Both classical and engineered phage strategies face shared challenges. Phages often have a narrow host range, bacteria can evolve resistance rapidly, and manufacturing and regulatory pathways are still developing [205,206,209]. For CRKP, capsule diversity and rapid surface evolution further complicate phage design, so future clinical use is likely to depend on phage cocktails or modular phage libraries linked to rapid strain typing.

Overall, CRISPR-based targeting, anti-plasmid strategies, and phage-assisted delivery show that carbapenem resistance in *K. pneumoniae* can be manipulated experimentally rather than only observed. At the same time, each approach remains in an early stage, with significant barriers related to delivery, safety, and scalability. It is therefore realistic to view these tools as complements to, rather than replacements for, antibiotics and infection control.

These experimental strategies highlight how molecular tools may eventually influence resistance trajectories rather than simply document them. Building on this direction, the next section examines how diagnostics, genomics, and emerging surveillance approaches can be linked to support coordinated interventions across clinical, hospital, and population settings.

## 9. Future Perspectives: Toward Integrated Molecular Surveillance and Intervention

### 9.1. Resistome-Based Surveillance Concepts

Resistome-based surveillance suggests that CRKP should be monitored not only through clinical isolates but also through the broader pools of resistance genes present in hospitals and communities. Metagenomic sequencing of sewage and hospital wastewater can capture a wide range of antimicrobial resistance genes and mobile elements in a single run, including those carried by *K. pneumoniae* and related *Enterobacterales* [210,211]. These studies show that wastewater resistomes often mirror resistance patterns in local hospitals and can reveal high-risk genes and plasmids before they appear in routine microbiology.

At the same time, metagenomic and targeted sequencing approaches are being adapted for national and regional surveillance networks. Pilot programs using urban sewage and wastewater treatment plants demonstrate that systematic resistome profiling can track temporal trends, geographic differences, and the movement of mobile genetic elements carrying carbapenemase genes [212,213,214]. In principle, these methods could be linked with hospital-based CRKP data to create an early warning system capable of detecting rising carbapenemase prevalence, shifts in dominant plasmid backbones, or incursions of new resistance variants.

Looking ahead, resistome-based surveillance will depend on standardized sampling, shared bioinformatic pipelines, and clear thresholds for public health action. Current work in wastewater-based epidemiology and One Health antimicrobial resistance programs already points toward integrated platforms that combine clinical microbiology, environmental sampling, and genomic analysis within a single surveillance framework [215,216,217].

### 9.2. Integration of Diagnostics with Stewardship

Rapid molecular diagnostics for CRKP have the greatest impact when embedded within antimicrobial stewardship. Studies of Gram-negative bacteremia show that combining rapid resistance testing with structured stewardship review can shorten time to optimal therapy, reduce unnecessary broad-spectrum antibiotic use, and improve clinical outcomes [218,219]. The same principles apply to CRKP. When molecular assays for carbapenemase genes are linked to real-time reporting, stewardship teams can use these results to de-escalate therapy, avoid ineffective agents, and prioritize infection control actions such as isolation and contact precautions [220,221].

Effective integration requires clear diagnostic pathways. Laboratories need agreed rules for when to perform molecular assays on blood cultures, respiratory samples, and rectal swabs. Clear guidance is also needed on how results should be communicated to clinicians and infection control teams. Stewardship programs then need simple, actionable algorithms that translate identified carbapenemase genes into specific treatment and isolation decisions. Over time, linking diagnostic findings with antibiotic consumption and outcome data can help hospitals adjust empirical regimens and refine screening strategies for CRKP [219,222].

### 9.3. System-Level Intervention Frameworks

The molecular and diagnostic advances described in earlier sections fit naturally within broader genomic surveillance and intervention frameworks. International initiatives now promote routine whole genome sequencing of priority pathogens and carbapenem-resistant *Enterobacterales* for surveillance, outbreak investigation, and tracking of high-risk clones and plasmids [223,224,225]. Real-time sequencing, combined with curated resistance gene databases, can identify the spread of specific *CRKP* lineages. It can also detect the movement of key carbapenemase plasmids. Together, these approaches allow transmission to be mapped across wards, hospitals, and regions [226].

System-level frameworks increasingly follow a One Health logic. Genomic and metagenomic data from clinical isolates, hospital and municipal wastewater, livestock, and surface waters can be integrated. These data can show how carbapenemase genes and *Klebsiella pneumoniae* lineages move between human and environmental reservoirs [215,227,228]. National genomic strategies now outline how sequencing capacity, data sharing, and governance structures can be organized so that AMR insights inform policies on infection prevention, water management, and antibiotic use [229].

For CRKP, an integrated future model would align three levels. At the bedside, rapid molecular tests would guide initial management and isolation decisions. At the hospital level, sequencing and molecular screening data would support stewardship, outbreak control, and targeted ward-level interventions. At the population level, resistome-based and genomic surveillance would track high-risk genes and clones across healthcare facilities and communities. Coordinating these layers offers a practical path toward earlier detection, more targeted response, and gradual reduction in the clinical impact of carbapenem resistance in *K. pneumoniae*.

## 10. Conclusions

Carbapenem resistance in *K. pneumoniae* does not arise from a single gene or from isolated hospital outbreaks. Instead, it develops through continued antibiotic pressure, the easy sharing of resistance between bacteria, and the ability of this organism to persist in the human body, particularly in the gut. These factors explain why resistant strains spread efficiently and remain hard to control in hospital settings. A central conclusion of this review is that resistance in *K. pneumoniae* functions as a connected process. Some bacterial groups become widespread, resistance moves readily between bacteria, and gradual biological change helps reduce the cost of carrying resistance. The bacteria that normally live in the gut play an especially important role. They form a hidden reservoir where resistance can circulate for long periods without causing illness or being noticed. Rapid molecular tests have greatly improved the speed at which carbapenem-resistant *K. pneumoniae* can be identified, allowing earlier infection control measures and more informed antibiotic choices. Even so, these tests can miss small numbers of resistant bacteria, unseen genetic reservoirs, or newly emerging resistance, which allows the problem to continue quietly. Lasting progress will require coordination across multiple areas rather than isolated technical advances. Combining fast testing with careful antibiotic use and wider monitoring of resistance in patients and the surrounding environment offers the most realistic way forward. New approaches that aim to weaken resistance directly are encouraging, but at present they should be viewed as supportive tools alongside established infection prevention and public health practices.

## Figures and Tables

**Table 1:** Clinical applications of molecular diagnostics for CRKP.

Diagnostic Platform	Molecular Targets	Specimen and Clinical Setting	Clinical Application	Key Findings from Clinical Studies	References
Real-time PCR for *blaKPC* from blood cultures	*blaKPC*	Positive blood culture bottles from patients with suspected bloodstream infection	Early confirmation of blaKPC-producing *K. pneumoniae* to guide rapid therapy decisions	Detected *blaKPC* with 100% sensitivity and specificity; limit of detection about 10–20 CFU per reaction	[172]
Multiplex real-time PCR for epidemic CRKP clones	*blaKPC* and ST258-associated markers	Cultured *Klebsiella pneumoniae* isolates during hospital outbreaks	Rapid identification of high-risk blaKPC-producing ST258 *K. pneumoniae*	100% sensitivity and specificity in tested isolates; faster than MLST	[173]
Xpert Carba-R NAAT	*blaKPC, blaNDM, blaVIM, blaOXA-48-like, blaIMP*	Rectal swabs and clinical isolates in screening programs	Rapid detection and classification of carbapenemase genes for infection control	Sensitivity about 93–98% and specificity close to 100% across multiple evaluations	[145,174,175]
LAMP assays	*K. pneumoniae* markers and *blaKPC* or *blaNDM*	Cultured isolates and selected clinical samples	Rapid detection of CRKP without thermocyclers	Results within about 60 min with high concordance to PCR	[149,153,154]
CRISPR–Cas13a assays	*blaKPC* and *blaNDM*	Clinical isolates and simulated clinical samples	Highly sensitive detection of CRKP with portable formats	Detection limits down to a few copies of DNA with full agreement to qPCR	[157,163]

## Data Availability

Not applicable.
